# IgG4-Related Prostatitis Detected by Whole-Body Diffusion-Weighted Imaging With Background Body Signal Suppression (DWIBS)

**DOI:** 10.7759/cureus.103883

**Published:** 2026-02-18

**Authors:** Hajime Kido, Terumi Kamisawa, Keigo Setoguchi, Shin-ichiro Horiguchi, Fumitaka Koga

**Affiliations:** 1 Department of Urology, Tokyo Metropolitan Cancer and Infectious Diseases Center Komagome Hospital, Tokyo, JPN; 2 Department of Gastroenterology, Tokyo Metropolitan Cancer and Infectious Diseases Center, Komagome Hospital, Tokyo, JPN; 3 Department of Rheumatology, Komagome Hospital, Tokyo, JPN; 4 Department of Pathology, Tokyo Metropolitan Cancer and Infectious Diseases Center, Komagome Hospital, Tokyo, JPN; 5 Department of Urology, Tokyo Metropolitan Cancer and Infectious Diseases Center, Komagome Hospital, Tokyo, JPN

**Keywords:** diffusion-weighted imaging with background body signal suppression, igg4, igg4-related disease, prostate biopsy, prostatitis

## Abstract

IgG4-related disease (IgG4-RD) is a systemic fibro-inflammatory disorder that can affect multiple organs, but involvement of the prostate is rare. Most reported cases of IgG4-related prostatitis are associated with multiorgan disease and present with lower urinary tract symptoms (LUTS). Herein, we present a rare case of IgG4-related prostatitis, which was detected by whole-body diffusion-weighted imaging with background body signal suppression (DWIBS) and was pathologically diagnosed by MRI-fusion target biopsy, accompanied by IgG4-related dacryoadenitis/sialadenitis and multiple lymphadenopathies. Oral prednisolone relieved symptoms including general malaise, weight loss, and LUTS, with decreases in serum IgG4 levels. At the same time, DWIBS showed a remarkable signal reduction in the prostate and other involved organs. The present case suggests the usefulness of DWIBS in detecting and monitoring IgG4-RD including IgG4-related prostatitis.

## Introduction

IgG4-related disease (IgG4-RD) is an immune-mediated and fibro-inflammatory disease characterized by elevation of serum IgG4 levels and enlargement or mass-formation in various organs. It usually occurs in elderly men. Histopathological features of IgG4-RD are abundant infiltration of lymphocytes and plasma cells, storiform fibrosis, and obliterative phlebitis [1,2]. According to the 2020 revised comprehensive diagnostic criteria for IgG4-RD [3], one of the pathological diagnostic items is the number of IgG4-positive plasma cells greater than 10 per high-powered field and the ratio of IgG4-positive plasma cells/IgG-positive cells greater than 40%. IgG4-RD is a systemic condition that can involve any organ or site synchronously or metachronously [1,2]. 

IgG4-related prostatitis is a rare manifestation of IgG4-RD and was first reported by Yoshimura in 2006 [4]. Since then, it has been scantly reported in the literature. According to the cohort study [5] of the IgG4-RD involving the prostate, most patients with IgG4-related prostatitis present with lower urinary tract symptoms (LUTS) and are pathologically diagnosed by core-needle biopsies. In almost all cases of IgG4-related prostatitis, it is a manifestation of multiorgan-involving IgG4-RD. IgG4-related prostatitis is sometimes detected through imaging studies to check other organ involvement, such as MRI, CT, and 18F-fluorodeoxyglucose positron emission tomography (FDG-PET), in patients with IgG4-RD. Here, we describe a case of IgG4-related prostatitis diagnosed with the help of whole-body diffusion-weighted imaging with background body signal suppression (DWIBS). DWIBS enables three-dimensional observation for reconstruction due to imaging in the transverse plane and has been used mainly for identifying primary and metastatic lesions of malignant tumors [6,7].

## Case presentation

**Figure 1:**
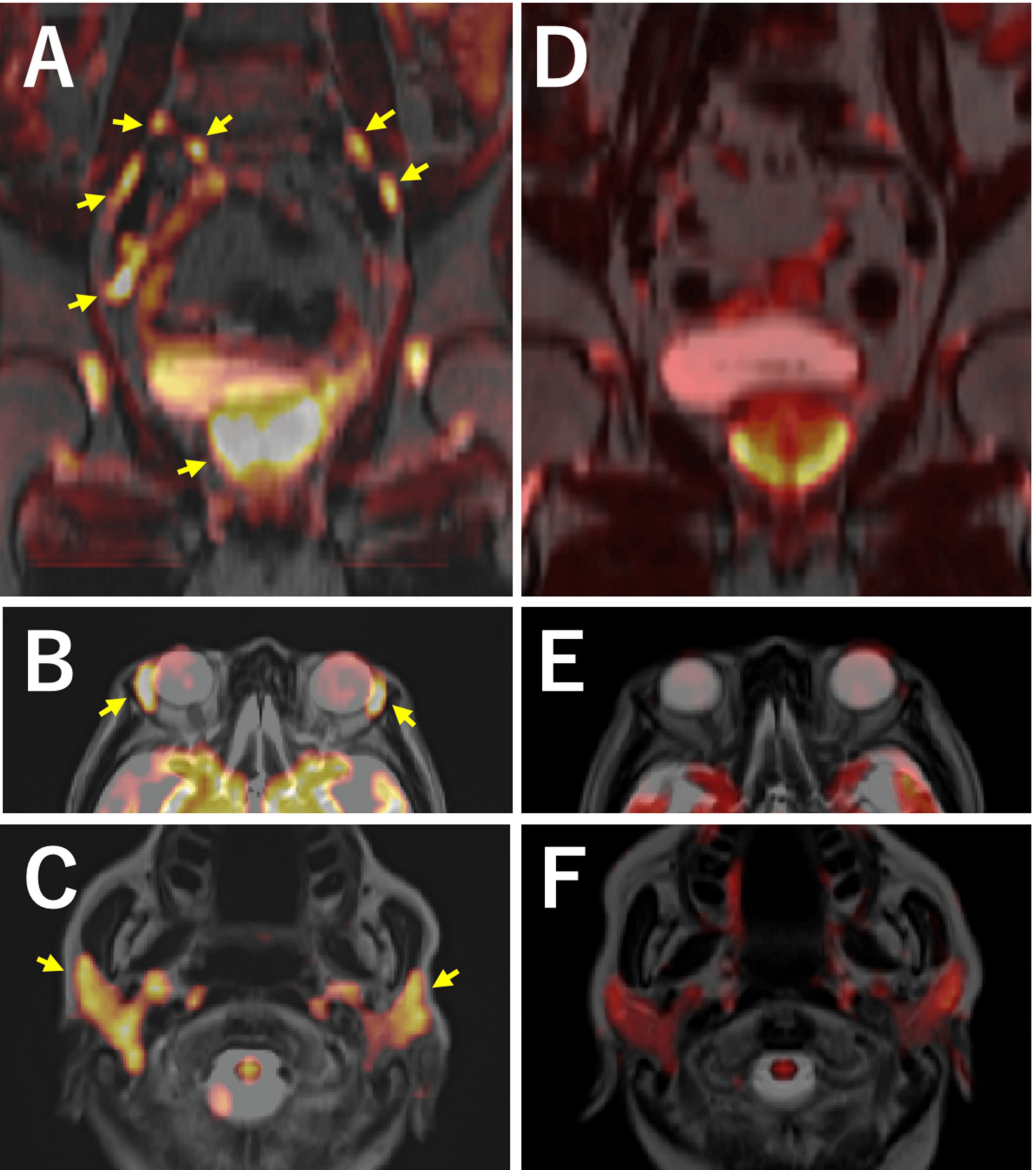
Whole-body DWIBS before (A-C) and after prednisolone therapy (D-F). The prostate, pelvic lymph nodes (A, coronal section, arrows), lacrimal glands (B, axial section, arrows), and submandibular glands (C, axial section, arrows) show high-signal intensity at diagnosis. Six months after prednisolone therapy, these lesions show a marked reduction in signal intensity (D-F).

**Figure 2:**
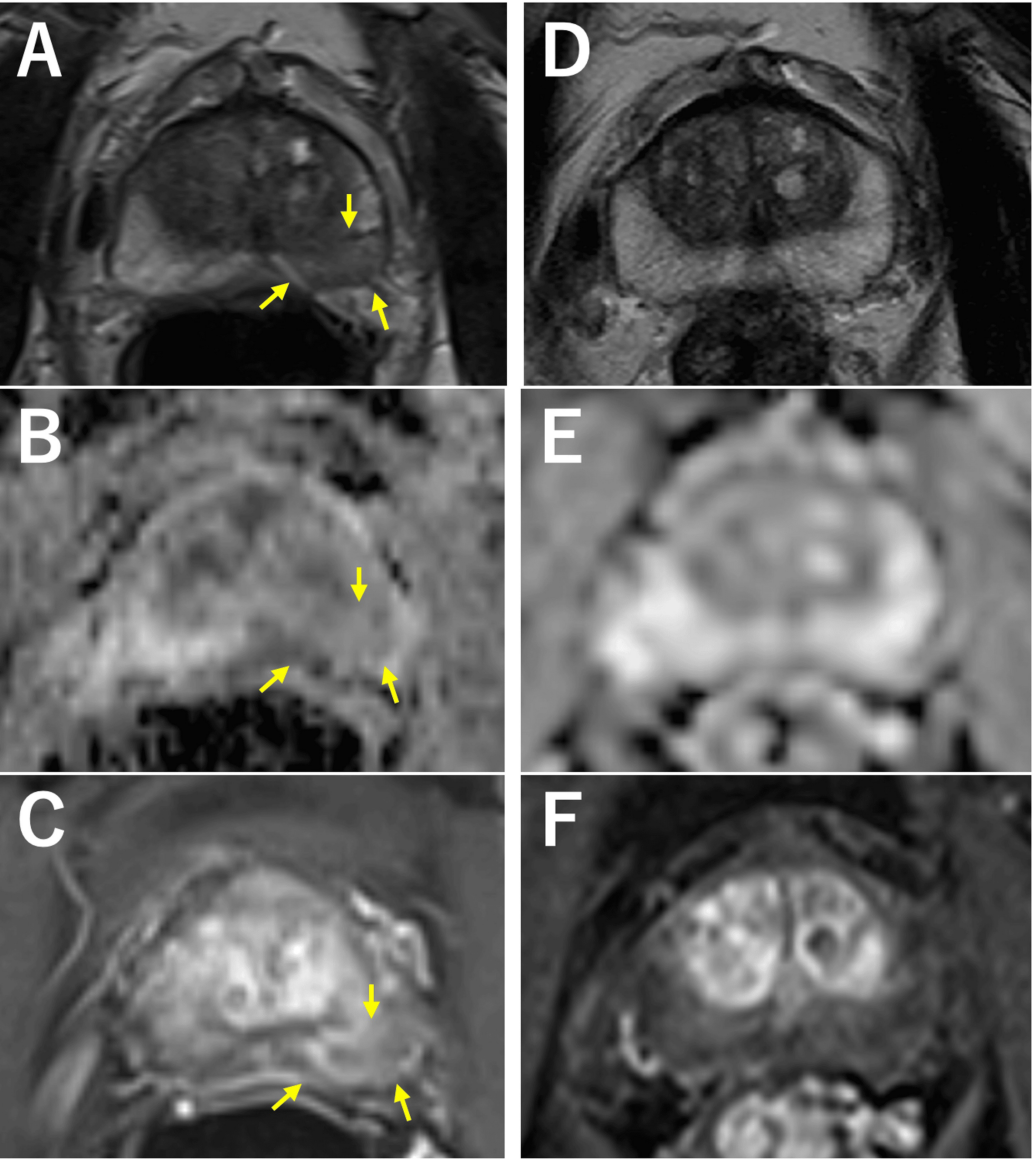
Multiparametric MRI of the prostate before (A-C) and after prednisolone therapy (D-F). Arrows indicate one of the inflammatory lesions exhibiting low-signal intensity on T2-weighted imaging (A) and apparent diffusion coefficient map (B) and slight enhancement on dynamic contrast enhanced imaging (C) at diagnosis. These inflammatory findings disappear after prednisolone therapy (D-F).

**Figure 3:**
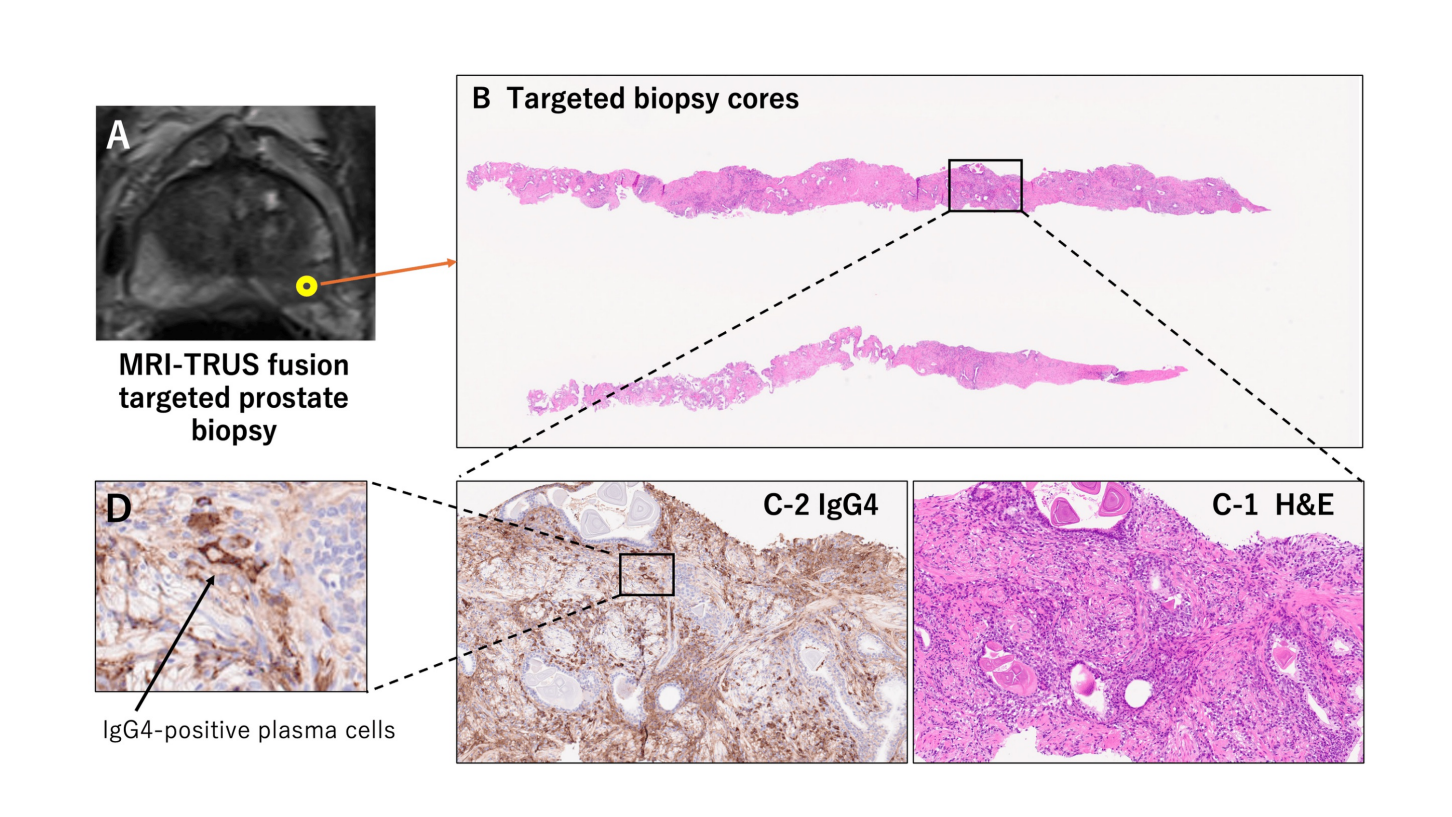
T2-weighted prostate MRI showing the inflammatory lesion (A), histopathology of MRI-fusion targeted biopsy cores (B), hematoxylin and eosin (H&E) staining (C-1), IgG4 immunostaining (C-2), and the corresponding high-power image (D). Histologically, inflammation is characterized by lymphoplasmacytic infiltrates. Immunohistochemical staining demonstrates abundant IgG4-positive plasma cells. TRUS: Transrectal ultrasound

A 76-year-old Japanese man was referred to the center of IgG4-RD at our hospital due to general malaise, weight loss, and a markedly elevated serum IgG4 level at 1,553 mg/dL (reference range: 11-121 mg/dL). He had swollen lacrimal and submandibular glands. Whole-body DWIBS, taken for systemic evaluation of inflammatory masses, revealed multiple high-signal lesions in the prostate along with multiple lymph nodes, lacrimal glands, and submandibular glands (Figures 1A-1C). He had moderate LUTS with an International Prostate Symptom Score (IPSS) of 13 and a slightly elevated serum prostate-specific antigen (PSA) level of 2.84 ng/mL. Multiparametric MRI of the prostate, taken for further assessment of intra-prostatic DWIBS-positive lesions, showed one cancerous and three inflammatory lesions (Figures 2A-2C). He underwent transperineal MRI-fusion target (two biopsy cores for each cancerous or inflammatory lesion, a total of eight cores) and 14-core systematic prostate biopsy. Histopathological examination revealed inflammation characterized by lymphoplasmacytic infiltrates, which were dense in some parts while sparse in other parts, in cores taken from the three inflammatory lesions on MRI. Immunohistochemical staining demonstrated abundant IgG4-positive plasma cells (>30 cells/high-power field), with an IgG4/IgG-positive cell ratio exceeding 50% (Figure 3). Storiform fibrosis or obliterative phlebitis was not observed. This case fulfilled diagnostic criteria of IgG4-RD according to the 2020 revised comprehensive diagnostic criteria (Appendix 1) [3] and the 2019 American College of Rheumatology/European League Against Rheumatism (ACR/EULAR) classification criteria (Appendix 2) [8]. Adenocarcinoma of grade group 2 with maximal cancer core length <1 mm was found only in three out of 14 systematic biopsy cores. He was placed on active surveillance for favorable intermediate-risk localized prostate cancer.

The patient started oral prednisolone at an initial dose of 27.5 mg/day, which was tapered to 10 mg over six months. He improved his fatigue levels and gradually gained weight. Six months after the treatment, whole-body DWIBS showed a remarkable reduction of signal intensity in the prostate as well as lymph nodes, lacrimal glands, and submandibular glands (Figures 1D-1F) with a reduced serum IgG4 level of 586 mg/dL. His LUTS also improved with an IPSS of six in parallel with PSA reduction to 0.87 ng/mL. Inflammatory lesions almost disappeared on multiparametric MRI (Figures 2D-2F). Despite adding mycophenolate mofetil for subsequent increases in serum IgG4 levels, his symptoms have been well controlled with prednisolone of 5 mg/day and mycophenolate mofetil of 750 mg/day 18 months after the initiation of the treatment. 

## Discussion

The present case is unique in that positive findings on DWIBS prompt the diagnosis of IgG4-related prostatitis, which is histologically made by MRI-fusion target biopsy. This case also illustrates that DWIBS, along with multiparametric MRI, reflects resolution of IgG4-related prostatitis in response to treatment. 

IgG4-RD is a systemic condition that affects various organs with organomegaly or hypertrophy in a simultaneous or metachronous manner. Although it can involve any sites or organs, including the pancreas, salivary and lacrimal glands, kidney, bile duct, and retroperitoneum, involvement of the prostate is rare [1,2,9]. Since the first report of IgG4-related prostatitis in 2006 [4], only 16 case reports and three case series have been published [5,10,11]. Approximately 80% of patients with IgG4-related prostatitis presented with LUTS such as urinary frequency, dysuria, or urinary retention, and they were usually diagnosed by core-needle prostate biopsy to differentiate from prostate cancer or by transurethral resection of the prostate to treat lower urinary tract obstruction [5]. IgG4-related prostatitis frequently involves multiple extra-prostatic organs.

The present patient complained of weight loss and general malaise with elevated serum IgG4 levels, and whole-body DWIBS was performed on suspicion of IgG4-RD. DWIBS revealed high signal intensity in the prostate and multiple lymph nodes, along with lacrimal and submandibular glands. Histology of the prostatic biopsy fulfilled diagnostic criteria by the 2020 revised comprehensive diagnostic criteria for IgG4-RD (Appendix 1) [3]. According to the 2019 ACR/EULAR classification criteria of IgG4-RD (Appendix 2) [8], the total point was 29, which surely met the classification criteria for IgG4-RD. Improvement of symptoms and serum IgG4 levels, and findings on subsequent MRI and DWIBS after steroid therapy strongly support the diagnosis of IgG4-related prostatitis. 

FDG-PET is a valuable tool for the diagnosis and staging of several types of malignancies. As FDG uptake is also observed at inflammatory sites, efficacy of FDG-PET for the diagnosis of IgG4-RD was reported [12,13]. Several cases of IgG4-related prostatitis were diagnosed with the help of FDG-PET [14,15]. However, FDG-PET has various drawbacks such as radiation exposure, prolonged period of time of examination and high cost. DWIBS functions by exploiting differences in water molecule diffusion, yielding high-signal intensity in highly cellular lesions or tissues with restricted diffusion [6,7]. Despite being originally developed in the oncology field, DWIBS can also demonstrate a high signal in inflammatory lesions [16]. Whole-body DWIBS could be a simple, non-invasive, and useful imaging study to systemically assess organs involved in IgG4-RD.

## Conclusions

We reported a rare case of IgG4-related prostatitis that was diagnosed on the basis of whole-body DWIBS findings and was pathologically confirmed by MRI-TRUS fusion targeted biopsy of inflammatory prostatic lesions. The diagnostic process would be unique in this case and underscore the diagnostic value of DWIBS. It also depicted the improvement of symptoms of IgG4-RD including prostatitis after corticosteroid therapy, indicating the role of DWIBS in monitoring the disease.

Given the rarity of reported cases, further accumulation of cases of IgG4-related prostatitis is essential to better define its clinical spectrum, establish optimal diagnostic strategies, and clarify long-term outcomes. The role of DWIBS in detecting and monitoring IgG4-related prostatitis should also be evaluated in future studies.

## Appendices

Appendix 1

**Table 1:** The 2020 revised comprehensive diagnostic (RCD) criteria for IgG4-RD. IgG4-RD: IgG4-related disease

[Item 1]	clinical and radiological features	
	One or more organs show diffuse or localized swelling or a mass or nodule characteristic of IgG4-RD. In single organ involvement, lymph node swelling is omitted.	
[Item 2]	serological diagnosis	
	Serum IgG4 levels greater than 135 mg/dl.	
[Item 3]	pathological diagnosis	
	Positivity for two of the following three criteria:	
	①	Dense lymphocyte and plasma cell infiltration with fibrosis.
	②	Ratio of IgG4-positive plasma cells /IgG-positive cells greater than 40% and the number of IgG4-positive plasma cells greater than 10 per high powered field
	③	Typical tissue fibrosis, particularly storiform fibrosis, or obliterative phlebitis.
Diagnosis:	Definite:	1) +2) +3)
	Probable:	1) +3)
	Possible:	1) +2)

Appendix 2

**Table 2:** 2019 ACR/EULAR classification criteria for IgG4-related disease

Step1：	Entry criteria						
	At least one organ involvement characteristic of IgG4-related disease (IgG4-RD) among the 11 predefined organ sites, based on clinical and/or radiologic evidence.						
Step 2:	Exclusion Criteria						
	Diseases characterized by fever, steroid resistance, or specific autoantibodies must be excluded based on clinical, serologic, and pathologic findings.						
Step 3:	Inclusion Criteria (Weighted Scoring System)						
	A total score of ≥20 points is required to fulfill the classification criteria.						
	Histopathology						
		Finding	Score				
		Dense lymphocytic infiltration	4				
		Lymphocytic infiltration with obliterative phlebitis	6				
		Lymphocytic infiltration with storiform fibrosis	13				
	Immunohistochemistry						
		IgG4-positive plasma cell / IgG-positive plasma cell ratio and number of IgG4-positive plasma cells					
				IgG4＋cells／HPF			
				0～9	Indeterminate	10～50	≧51
		IgG4＋／IgG＋cells ratio	0～40	0	7	7	7
			Indeterminate	0	7	7	7
			41～70	7	7	14	14
			≧71％	14	7	14	16
	Serum IgG4 Level						
		Serum IgG4 concentration	Score				
		Normal or not tested	0				
		Above normal to <2× upper limit of normal	4				
		≥2× to <5× upper limit of normal	6				
		≥5× upper limit of normal	10–11				
	Bilateral Lacrimal / Salivary Gland Enlargement						
		Finding	Score				
		Enlargement of 1 gland set	6				
		Enlargement of ≥2 gland sets	14				
	Thoracic Involvement						
		Finding	Score				
		Bronchovascular bundle thickening	4				
		Paravertebral band-like soft tissue thickening	10				
	Pancreatic / Biliary Involvement						
		Finding	Score				
		Diffuse pancreatic enlargement	8				
		Pancreatic enlargement with capsule-like rim	11				
		Pancreatic enlargement with capsule-like rim and biliary involvement	19				
	Renal Involvement						
		Finding	Score				
		Hypocomplementemia	6				
		Renal pelvic wall thickening	8				
		Bilateral renal cortical low-density lesions	10				
	Retroperitoneal Involvement						
		Finding	Score				
		Diffuse periaortic soft tissue thickening	4				
		Soft tissue surrounding the infrarenal aorta or iliac arteries	8				
Step 4:	Total Inclusion Points						
	IgG4-related disease (IgG4-RD) classification criteria are fulfilled when the sum of the eight inclusion items in Step 3 is ≥20 points.						
